# Global Burden of Ischemic Heart Disease and Ischemic Stroke, 1990–2023: Spatial Heterogeneity, SDI Inequalities, Pandemic‐Era Inflections, and Scenario Forecasts to 2035 Using GBD 2023

**DOI:** 10.1155/crp/6248551

**Published:** 2026-09-15

**Authors:** Haohao Chen, Boya Hu, Ziwen Xiong, Mengting Zhao, Bingxuan Chen, Jinhua Yang, Yali Wang, Ying Zeng, Fenfei Gao, De Cai

**Affiliations:** ^1^ Department of Pharmacy, The First Affiliated Hospital of Shantou University Medical College, Shantou, China, stuh.com.cn; ^2^ Department of Pharmacology, Shantou University Medical College, Shantou, China, stu.edu.cn; ^3^ Shantou Key Laboratory of Precision Medicine and Intelligent Evaluation, Shantou, China; ^4^ Department of Neurosurgery, The First Affiliated Hospital of Shantou University Medical College, Shantou, China, stuh.com.cn

**Keywords:** age-standardized rates, COVID-19, global burden of disease, ischemic heart disease, ischemic stroke, sociodemographic index

## Abstract

**Background:**

Ischemic heart disease (IHD) and ischemic stroke (IS) remain leading causes of death and disability, with substantial geographic and sociodemographic inequalities. We assessed long‐term trends, sociodemographic index (SDI) disparities, COVID‐19–period inflections, risk‐factor profiles, and scenario forecasts using Global Burden of Disease (GBD) 2023 estimates.

**Methods:**

Using GBD 2023 data (1990–2023), we analyzed age‐standardized incidence (ASIR), death (ASDR), and DALY rates (ASDALY) globally and across SDI quintiles. COVID‐19 impacts were evaluated using segmented log‐linear models (2010–2019 vs. 2020–2023), estimating the average annual percent change (AAPC) and counterfactual deviations extrapolating pre‐COVID trends. Risk attribution used the GBD comparative risk assessment framework. ARIMA‐based forecasts projected global ASDR/ASDALY to 2035, with S1 fitted to 2010–2019 and S2 to 2010–2023; 1990‐start forecasts were assessed as sensitivity analyses.

**Results:**

In 2023, ASDRs varied widely across countries (IHD 24.9–441.0; IS 6.0–182.5 per 100,000). From 1990 to 2023, global ASDR declined for both diseases (IHD −38.2%; IS −53.1%), with an IHD SDI reversal (high SDI −52.8% vs. low SDI +13.4%). During 2020–2023, IHD mortality plateaued (AAPC 0.06%/year), whereas IS continued to decline but slowed (AAPC −1.48%/year). Forecasts to 2035 indicated a higher projected burden under S2 than S1.

**Conclusions:**

Progress in IHD and IS burden has been uneven, with widening SDI disparities and pandemic‐era attenuation, underscoring the need for equitable and resilient prevention and care.

## 1. Introduction

Ischemic heart disease (IHD) and ischemic stroke (IS) remain among the leading contributors to premature mortality and long‐term disability worldwide. Their trajectories are shaped by population aging, evolving cardiometabolic risk exposures, and unequal access to effective prevention and treatment [1, 2]. Contemporary Global Burden of Disease (GBD) syntheses continue to document substantial geographic and development‐related heterogeneity in cardiovascular burden, underscoring the need for updated comparative assessments to inform priority setting and resource allocation [2, 3].

Although prior GBD analyses have provided essential insights into long‐term changes in age‐standardized rates, gaps remain for policy‐relevant interpretation. First, IHD and IS are often reported in parallel but are less frequently compared systematically across multiple metrics—incidence, mortality, and disability‐adjusted life years (DALYs)—within a unified framework that simultaneously addresses geography, sociodemographic development, and demographic stratification. Second, recent GBD stroke‐specific work has highlighted that improvements in stroke mortality and DALY rates can outpace changes in incidence, raising the question of whether analogous or divergent patterns apply to IHD when assessed side‐by‐side [4–6].

The COVID‐19 pandemic adds a further dimension of urgency. Beyond its direct clinical consequences, the pandemic plausibly influenced cardiovascular burden through disruptions in chronic disease management and medication continuity, delayed emergency presentations, constrained acute‐care capacity, and broader behavioral and socioeconomic shocks. Empirical studies have reported marked reductions in hospital encounters for acute myocardial infarction and changes in treatment and outcomes during periods of high COVID‐19 hospital burden, alongside global declines in stroke admissions and reperfusion‐related activity such as mechanical thrombectomy [7–9]. In parallel, cohort‐based evidence indicates increased post‐acute cardiovascular risk following SARS‐CoV‐2 infection, and population‐level analyses have identified excess cardiovascular mortality peaks across pandemic waves [10, 11]. Together, these observations motivate analytic approaches that can quantify whether pandemic‐era trajectories deviated from prepandemic trends and whether such deviations differed between IHD and IS.

Prevention priorities therefore warrant renewed attention. Recent GBD risk‐factor assessments highlight the persistent contribution of modifiable behavioral, metabolic, and environmental risks—such as tobacco exposure, unhealthy dietary patterns, and air pollution—while emphasizing that risk profiles and their attributable contributions vary substantially by region and sex [6]. High‐impact prevention guidance increasingly supports policy‐ and environment‐oriented approaches (e.g., healthier food environments and comprehensive tobacco control), alongside equitable scale‐up of evidence‐based cardiovascular prevention and long‐term management [12–14].

We used GBD 2023 estimates to provide an integrated assessment of IHD and IS burden from 1990 to 2023. Specifically, we (i) characterized country‐level spatial heterogeneity in 2023; (ii) quantified long‐term trends and sociodemographic inequalities across sociodemographic index (SDI) quintiles; (iii) described age‐ and sex‐specific mortality patterns; (iv) evaluated pandemic‐era inflections using segmented trend analyses and counterfactual deviations; (v) examined regional and sex heterogeneity in leading attributable risks and shifts in global risk profiles between pre‐COVID and COVID periods; and (vi) generated scenario‐based forecasts of global age‐standardized death rates (ASDR) and age‐standardized DALY rates (ASDALY) through 2035 to inform planning under alternative postpandemic trajectories.

## 2. Method

### 2.1. Study Design and Data Sources

This ecological, longitudinal analysis quantified the global burden of IHD and IS from 1990 to 2023, with a specific focus on spatial heterogeneity in 2023, long‐term trends, sociodemographic inequalities, COVID‐19–period deviations, risk‐factor attribution patterns, and scenario‐based forecasting through 2035. Country‐level and aggregate estimates were obtained from the GBD 2023 study results database (Institute for Health Metrics and Evaluation, IHME) [1, 2]. We extracted annual estimates by location (countries/territories and global aggregates), sex, and age group for relevant measures and metrics as described below. As analyses relied exclusively on publicly available, de‐identified, aggregated GBD outputs, no individual‐level data were used. Case definitions for IHD and IS followed the GBD 2023 standard, which are based on ICD‐10 codes I20‐I25 for IHD and I63, I64, and H34.1 (excluding I63.6) for IS.

### 2.2. Outcomes, Measures, and Standardization

We analyzed three core age‐standardized metrics for each cause (IHD and IS): (1) age‐standardized incidence rate (ASIR); (2) age‐standardized death rate (ASDR); and (3) ASDALY, all per 100,000 population. Age standardization followed the GBD standard population and GBD modeling conventions [1]. For each estimate, we used the point estimate and corresponding 95% uncertainty interval (UI) (lower and upper bounds) as provided by GBD. YLDs in the GBD 2023 framework are estimated using a prevalence‐based approach (standard since GBD 2010), meaning that YLDs reflect nonfatal health loss from existing (prevalent) cases rather than future loss from new (incident) cases. To contextualize age‐standardized trends, we also extracted global all‐age deaths and DALYs (metric: number) for IHD and IS for 1990–2023; selected reference years and 1990–2023 percentage changes were summarized using unrounded GBD point estimates.

### 2.3. Sociodemographic Development Stratification

To assess development‐related inequalities, analyses were stratified by SDI. SDI quintiles (low, low‐middle, middle, high‐middle, and high) were defined using the GBD SDI framework [2]. We compared levels and trends across quintiles for 1990–2023 and summarized disparities using both absolute levels in reference years and relative changes over time.

### 2.4. Spatial Analyses in 2023

To characterize geographic heterogeneity in contemporary burden, we mapped country‐level ASDRs in 2023 for IHD and IS using choropleth visualization. Countries/territories were grouped into ordered categories to highlight clustering of high‐ and low‐burden settings. Summary statistics (minimum, maximum, and distributional patterns) were calculated to compare upper‐end burden between IHD and IS. Spatial analyses were descriptive and intended to contextualize regional clustering rather than infer causality.

### 2.5. Long‐Term Trends and Reference‐Year Comparisons (1990–2023)

We quantified temporal trends globally and by SDI quintile using annual time series (1990–2023) for ASIR, ASDR, and ASDALY. Long‐term change was summarized using:

Percent change (1990–2023):

(1) %Δ19902023−=Rate2023−Rate1990Rate1990×100.

Percent change (2019–2023) to capture the immediate pre‐/peri‐pandemic era:

(2) %Δ20192023−=Rate2023−Rate2019Rate2019×100.

We extracted reference‐year estimates (1990, 2019, 2020, and 2023) by SDI quintile to facilitate standardized cross‐sectional comparisons and to summarize long‐term and recent changes.

### 2.6. Age‐ and Sex‐Specific Analyses

To describe demographic patterning, we extracted age‐specific death rates for males and females in 1990 and 2023 and summarized age gradients using prespecified aggregated age bands (e.g., 15–49, 50–74, and ≥ 75 years). We then calculated proportional changes between 1990 and 2023 within each age–sex stratum to identify groups with the largest relative declines. These analyses were descriptive and aimed to characterize age concentration of mortality burden and sex differences across age strata.

### 2.7. COVID‐19 Period Impact: Segmented Slopes and Counterfactual Deviations

We evaluated pandemic‐period impact using two complementary approaches, defining pre‐COVID as 2010–2019 and the COVID period as 2020–2023.

#### 2.7.1. Segmented Trend Analysis (Average Annual Percent Change [AAPC])

To quantify slope changes across periods, we fit segmented log‐linear models to annual global rates (ASDR and ASDALY) with a breakpoint at 2020, estimating the AAPC within each segment [15]. Specifically, rates were modeled on the log scale to approximate constant proportional change over time, and AAPC was derived from the segment‐specific slope:

(3) AAPC=eβ−1×100%,

where *β* is the estimated annual slope from the log‐linear model. The corresponding 95% confidence intervals (CIs) for AAPC were computed using standard errors from the fitted regression. Parallel analyses were performed within SDI quintiles to assess heterogeneity in slope changes across development strata.

#### 2.7.2. Observed vs. Expected Counterfactual Deviations

We constructed counterfactual trajectories by extrapolating pre‐COVID (2010–2019) trends into 2020–2023, generating expected values for each year and comparing them with observed GBD estimates [15, 16]. Year‐specific and mean deviations were summarized as relative gaps:

(4) Gapt=Observedt−expectedtExpectedt×100%.

We reported both the mean relative gap across 2020–2023 and the year‐by‐year gaps, enabling assessment of whether observed trajectories converged back toward the counterfactual after 2020 and whether patterns differed between IHD and IS and between ASDR and ASDALY.

### 2.8. Risk‐Factor Attribution and Profile Shifts

Risk‐factor patterns were evaluated using the GBD comparative risk assessment framework, which provides cause‐specific burden attributable to individual risks. We focused on attributable burden for ASDALY and summarized the following:

Regional and sex heterogeneity in 2023: We identified and ranked leading risks by attributable DALY rate within each GBD region and separately for males and females; we additionally reported the corresponding attributable DALY percentage to characterize relative contributions.

Global risk‐profile shift (pre‐COVID vs. COVID period): We compared the relative composition of attributable DALYs between 2010–2019 and 2020–2023. For each risk, we calculated the percentage‐point change (Δpp) in attributable DALY percentage:

(5) Δpp=Share20202023−−Share20102019−,

where share denotes the attributable DALY percentage. This approach isolates compositional changes (risk ranking and contribution share) rather than changes in absolute attributable burden alone.

### 2.9. Forecasting Through 2035: Autoregressive Integrated Moving Average (ARIMA)‐Based Scenario Projections

We projected global ASDR and ASDALY for IHD and IS through 2035 using ARIMA models fitted to annual time series. Forecasting was implemented under three scenarios:

S1 (no‐pandemic counterfactual): Models were fitted to pre‐COVID data (2010–2019) and extrapolated forward to 2035 to represent the continuation of prepandemic trajectories.

S2 (pandemic‐influenced continuation): Models were fitted to the full series 2010–2023, incorporating the pandemic period into projected trajectories.

S3 (recovery): Projections were parameterized to transition gradually from the S2 trajectory toward S1 over a prespecified recovery window starting in 2024, converging with S1 thereafter.

Model specification followed standard ARIMA procedures, including assessment of stationarity, differencing as required, and selection of autoregressive and moving‐average orders using information criteria [17]. Forecast uncertainty was summarized using 95% prediction intervals; lower bounds were constrained to be non‐negative where applicable. Scenario separation was evaluated by comparing projected levels in 2030 and 2035 and by visually examining divergence of forecast trajectories after 2024. These projections were intended as conditional scenario trajectories rather than deterministic predictions. The 2010‐start models were treated as the primary analysis because they capture the more recent prepandemic trajectory and align with the pandemic‐period analyses, whereas 1990‐start models were used as sensitivity analyses to assess dependence on the training window and longer‐term secular trends. S1 and S2 were not intended to represent formal best‐ and worst‐case bounds.

### 2.10. Uncertainty Handling

We reported GBD‐provided 95% UIs for GBD estimates. Forecast uncertainty for ARIMA projections was summarized using 95% prediction intervals. For derived quantities (e.g., percent change and counterfactual gaps), we primarily computed values using point estimates; where stricter uncertainty propagation is required, the same calculations can be repeated across uncertainty draws (when available) to obtain empirical UIs. All uncertainty reporting followed a two‐sided 95% convention.

### 2.11. Statistical Software and Reproducibility

All analyses were conducted in R (Version 4.5.1; R Foundation for Statistical Computing, Vienna, Austria). Data management and visualization used a reproducible pipeline (data cleaning, aggregation, and figure/table generation scripts). Spatial mapping relied on standard geospatial workflows (country boundary shapefiles merged with GBD country estimates). Time‐series modeling and forecasting used established R time‐series packages (e.g., functions for ARIMA fitting, model fitting, and forecast interval generation). Final figures and tables were generated directly from the analytic pipeline to ensure reproducibility.

### 2.12. Patient and Public Involvement

No patients or members of the public were involved in the design, conduct, reporting, or dissemination plans of this research. This study used de‐identified, aggregated data from the GBD 2023 database, which does not include individual‐level patient data. Therefore, patient and public involvement was not applicable or feasible for this ecological, longitudinal analysis. Clinical trial number: not applicable.

## 3. Results

### 3.1. Spatial Patterns of Mortality Burden in 2023

**FIGURE 1:**
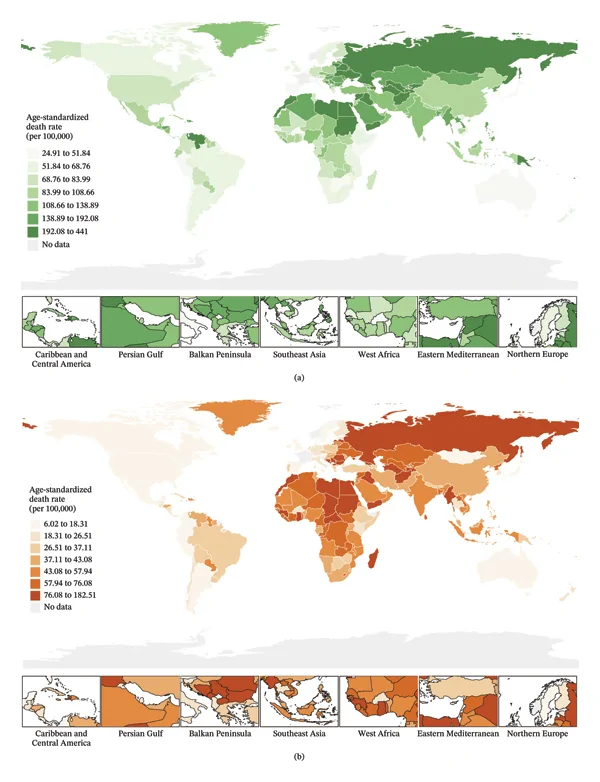
Global distribution of age‐standardized death rates (ASDRs) for ischemic heart disease and ischemic stroke in 2023. Country‐level ASDRs (per 100,000 population) in 2023 for both sexes are shown for (a) ischemic heart disease (IHD) and (b) ischemic stroke (IS). Darker shading indicates higher ASDRs; countries with missing estimates are shown in gray. Insets highlight areas with dense clusters of small countries/territories: the Caribbean and Central America, the Persian Gulf, the Balkan Peninsula, Southeast Asia, West Africa, the Eastern Mediterranean, and Northern Europe.

In 2023, substantial geographical heterogeneity was observed in country‐level ASDRs for both IHD and IS (Figure 1). For IHD, ASDRs ranged from 24.9 per 100,000 in the Republic of Korea to 441.0 per 100,000 in Egypt, with higher rates clustering in several parts of Central Asia/Eastern Europe and the Middle East and North Africa and additional high‐burden pockets in selected Pacific island nations. For IS, ASDRs ranged from 6.0 per 100,000 in Singapore to 182.5 per 100,000 in Afghanistan, with elevated mortality concentrated across parts of North Africa/Middle East and Eastern Europe, while comparatively lower rates were observed in many high‐income Asia–Pacific and Western European settings. Overall, the upper‐end mortality burden was higher for IHD than for IS (maximum ASDR 441.0 vs. 182.5 per 100,000).

### 3.2. Long‐Term Trends and SDI‐Related Inequalities in IHD and IS Burden (1990–2023)

**FIGURE 2:**
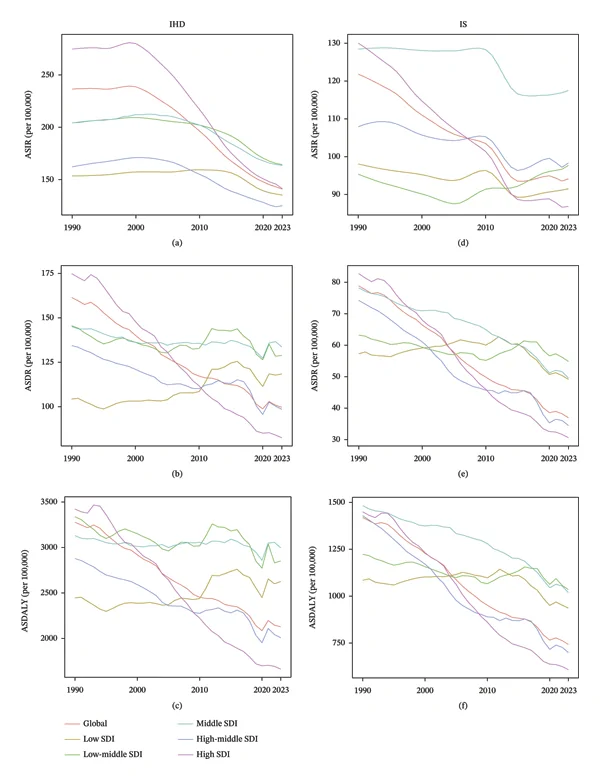
Long‐term trends in age‐standardized rates for ischemic heart disease and ischemic stroke by SDI quintiles, 1990–2023. Trends in age‐standardized incidence rate (ASIR), age‐standardized death rate (ASDR), and age‐standardized DALY rate (ASDALY) (per 100,000 population) for both sexes from 1990 to 2023 are shown for the global level and five sociodemographic index (SDI) quintiles. Panels show: (a) IHD ASIR; (b) IHD ASDR; (c) IHD ASDALY; (d) IS ASIR; (e) IS ASDR; and (f) IS ASDALY. Abbreviations: IHD, ischemic heart disease; IS, ischemic stroke; SDI, sociodemographic index.

From 1990 to 2023, age‐standardized rates generally declined for both IHD and IS, although the magnitude and SDI‐patterning differed by metric (Figure 2). Globally, IHD ASIR decreased from 236.5 to 140.9 per 100,000 (−40.4%), accompanied by reductions in IHD ASDR (161.4–99.7, −38.2%) and IHD ASDALY (3277.2–2128.6, −35.0%). In contrast, global IS rates showed a more modest decline in incidence (ASIR 121.8 to 94.1, −22.7%) but larger reductions in mortality and overall burden (ASDR 78.9 to 37.0, −53.1%; ASDALY 1419.5 to 742.3, −47.7%).

Marked SDI gradients were evident and evolved over time. For IHD, the high‐SDI quintile experienced the steepest long‐term decreases (e.g., ASDR −52.8% and ASDALY −51.4% from 1990 to 2023), whereas in the low‐SDI quintile IHD mortality and DALY rates increased over the same period (ASDR 104.4 to 118.4, +13.4%; ASDALY 2447.2 to 2626.6, +7.3%), resulting in a clear reversal of the early‐period pattern by 2023. For IS, mortality and DALY rates declined across all SDI strata, but reductions were largest in high SDI (ASDR −63.0%, ASDALY −58.1%) and comparatively smaller in lower SDI quintiles; IS incidence showed relative stagnation in several strata, including a slight increase in the low‐middle SDI quintile from 1990 to 2023 (+2.4%). Overall, these trajectories indicate substantial progress in high‐SDI settings and persistent SDI disparities, particularly for IHD. The underlying annual ASIR, ASDR, and ASDALY estimates at the global level and across SDI quintiles are provided in Supporting Table S1. Despite declining age‐standardized rates, absolute burden increased from 1990 to 2023: IHD deaths and DALYs rose by 60.6% and 51.5%, respectively, while IS deaths and DALYs increased by 27.6% and 29.1% (Supporting Table S12).

### 3.3. Key Reference Years and Percent Changes

ASDRs for 1990, 2019, 2020, and 2023, together with percentage changes over 1990–2023 and 2019–2023, are presented across SDI quintiles (Table 1). Globally, IHD ASDR declined from 161.41 per 100,000 in 1990 to 99.68 in 2023 (−38.24%), while IS ASDR fell from 78.85 to 36.95 (−53.14%). Over the post‐2019 period, global mortality changes were comparatively small in magnitude, with IHD decreasing by −1.70% and IS by −7.93% between 2019 and 2023.

**TABLE 1:** Age‐standardized death rates (ASDRs) for ischemic heart disease and ischemic stroke by SDI quintiles in 1990, 2019, 2020, and 2023, and percentage changes over 1990–2023 and 2019–2023.

Disease	SDI group	1990	2019	2020	2023	% change (1990–2023)	% change (2019–2023)
IHD	Global	161.41 (146.95–172.63)	101.40 (92.98–108.18)	98.80 (90.11–105.44)	99.68 (89.81–108.31)	−38.24 (−47.98 to −26.30)	−1.70 (−16.98–16.49)
	Low	104.39 (79.92–128.98)	115.90 (98.74–134.71)	111.39 (94.87–129.39)	118.42 (96.02–141.28)	13.44 (−25.55–76.78)	2.17 (−28.72–43.09)
	Low‐middle	145.53 (118.44–173.81)	129.52 (115.82–146.16)	126.34 (110.15–140.89)	128.88 (113.33–147.92)	−11.44 (−34.80–24.89)	−0.50 (−22.47–27.72)
	Middle	145.07 (122.71–168.34)	130.83 (117.97–143.35)	127.28 (113.71–139.78)	133.64 (117.92–149.21)	−7.88 (−29.95–21.60)	2.15 (−17.74–26.49)
	High‐middle	134.38 (118.21–149.76)	100.37 (91.12–107.49)	95.64 (85.89–102.81)	98.52 (87.28–107.55)	−26.69 (−41.72 to −9.01)	−1.85 (−18.80–18.04)
	High	174.86 (161.81–182.55)	86.26 (78.47–90.68)	85.11 (78.02–89.14)	82.50 (74.39–87.96)	−52.82 (−59.25 to −45.64)	−4.35 (−17.96–12.09)

IS	Global	78.85 (71.72–86.27)	40.13 (35.78–44.60)	38.58 (34.10–43.23)	36.95 (32.33–41.58)	−53.14 (−62.52 to −42.03)	−7.93 (−27.52–16.19)
	Low	57.29 (40.64–80.14)	52.62 (38.52–66.79)	50.73 (36.98–66.16)	49.15 (35.70–65.04)	−14.20 (−55.45–60.02)	−6.60 (−46.56–68.85)
	Low‐middle	63.23 (49.31–81.70)	58.42 (45.82–72.09)	56.62 (44.37–69.68)	54.85 (41.96–68.89)	−13.26 (−48.64–39.71)	−6.12 (−41.80–50.34)
	Middle	78.21 (64.19–92.05)	53.51 (44.71–64.13)	51.18 (41.96–61.77)	49.59 (38.98–58.93)	−36.59 (−57.65 to −8.19)	−7.33 (−39.21–31.80)
	High‐middle	74.24 (61.98–89.53)	38.02 (33.88–42.28)	35.33 (31.09–39.21)	34.49 (29.85–39.22)	−53.54 (−66.66 to −36.72)	−9.28 (−29.40–15.76)
	High	82.76 (76.32–88.01)	33.46 (29.68–35.92)	32.56 (28.89–34.73)	30.60 (26.84–33.52)	−63.02 (−69.51 to −56.08)	−8.56 (−25.28–12.92)

*Note:* Values are ASDRs per 100,000 population, with 95% uncertainty intervals (UIs) in parentheses. Percentage changes are reported as % (95% UI). Estimates are for both sexes. SDI quintiles (low, low‐middle, middle, high‐middle, and high) were defined using the GBD SDI framework; GBD, Global Burden of Disease.

Abbreviation: SDI, sociodemographic index.

Substantial SDI heterogeneity was observed, particularly for IHD. From 1990 to 2023, IHD ASDR decreased markedly in the high‐SDI quintile (174.86–82.50 per 100,000; −52.82%) but increased in the low‐SDI quintile (104.39–118.42; +13.44%). In contrast, IS ASDR decreased in all SDI strata, with the largest relative reduction in high SDI (82.76–30.60; −63.02%) and a more modest decline in low SDI (57.29–49.15; −14.20%). Patterns for overall burden were consistent (Supporting Table S2). Globally, IHD ASDALY declined from 3277.16 to 2128.58 (−35.05%) and IS ASDALY from 1419.46 to 742.32 (−47.70%); notably, IHD ASDALY increased in low SDI (+7.33%) but decreased substantially in high SDI (−51.42%).

At the country and territory level, the differential trajectories of IHD and IS were also evident. Based on GBD point estimates, IHD ASDR increased between 1990 and 2023 in 63 of 204 locations (30.9%), compared with 22 of 204 locations (10.8%) for IS; over 2019–2023, increases were observed in 110 locations (53.9%) for IHD versus 57 locations (27.9%) for IS. Corresponding ASDALY patterns were similar (65 vs. 23 locations with increases over 1990–2023 and 108 vs. 54 over 2019–2023). Country‐ and territory‐specific estimates for 1990, 2019, 2020, and 2023, together with percentage changes over 1990–2023 and 2019–2023, are provided in Supporting Tables S10 and S11.

### 3.4. Age‐ and Sex‐Specific Patterns

**FIGURE 3:**
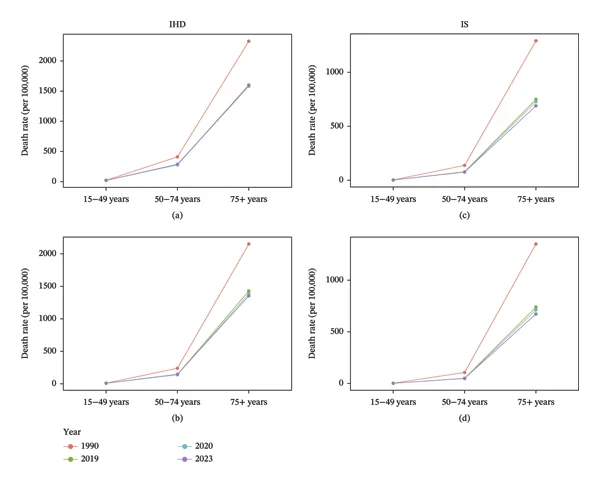
Age‐ and sex‐specific death rates for ischemic heart disease and ischemic stroke at the global level in 1990 and 2019–2023. Age‐specific death rates (per 100,000 population) for ischemic heart disease (IHD) and ischemic stroke (IS) are shown for males and females across three adult age groups (15–49 years, 50–74 years, and ≥ 75 years) in 1990, 2019, 2020, and 2023. Panels show: (a) IHD, males; (b) IHD, females; (c) IS, males; and (d) IS, females. Lines connect age‐group‐specific rates within each calendar year, and points indicate the observed values.

Age‐specific death rates rose sharply with advancing age in both IHD and IS, with the ≥ 75 years group consistently accounting for the highest mortality burden (Figure 3). In 2023, IHD death rates in the ≥ 75 years group reached 1585.9 per 100,000 in males and 1353.0 per 100,000 in females, compared with 287.3 and 146.9 per 100,000 in the 50–74 years group and 20.7 and 8.9 per 100,000 in the 15–49 years group, respectively. IS displayed a comparable age gradient but at lower absolute levels: In 2023, IS death rates in the ≥ 75 years group were 688.9 per 100,000 in males and 672.2 per 100,000 in females, while remaining much lower in younger adults (≤ 75.3 per 100,000 in 50–74 years and ≤ 1.7 per 100,000 in 15–49 years). When 2023 levels were evaluated against 1990, the largest proportional declines were concentrated in older adults, especially those aged ≥ 75 years (IHD: −31.8% in males and −37.1% in females; IS: −46.7% in males and −50.2% in females). By contrast, changes between 2019 and 2023 were comparatively modest across age–sex strata, indicating broadly stable recent patterns in age‐specific mortality. The underlying age‐ and sex‐stratified estimates are provided in Supporting Table S3.

### 3.5. COVID‐19 Period Impact: Slope Changes and Counterfactual Deviations

#### 3.5.1. Segmented Trends (AAPC)

**FIGURE 4:**
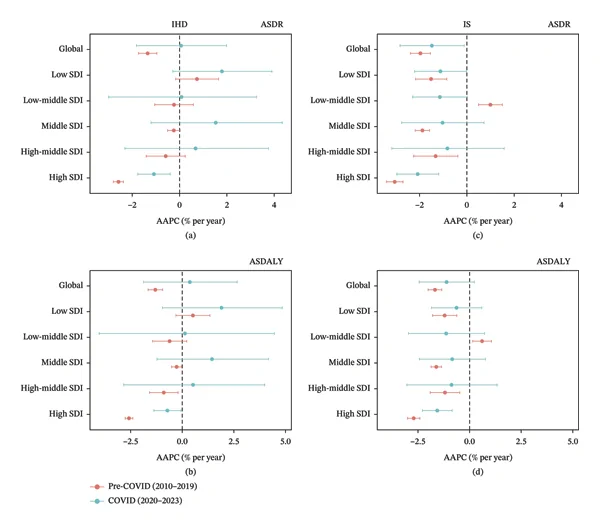
Segmented trends before and during the COVID‐19 period: AAPC in age‐standardized death and DALY rates. Average annual percent change (AAPC, % per year) was estimated for the pre‐COVID period (2010–2019) and the COVID period (2020–2023) for ischemic heart disease (IHD) and ischemic stroke (IS) at the global level and across SDI quintiles. Panels show: (a) AAPC in age‐standardized death rate (ASDR) for IHD; (b) AAPC in age‐standardized DALY rate (ASDALY) for IHD; (c) AAPC in ASDR for IS; and (d) AAPC in ASDALY for IS. Points represent AAPC estimates, and horizontal bars indicate 95% confidence intervals. The dashed vertical line denotes AAPC = 0. Abbreviations: AAPC, average annual percent change; SDI, sociodemographic index; ASDR, age‐standardized death rate; ASDALY, age‐standardized DALY rate.

Segmented trend analyses indicated a clear change in the slope of global burden trajectories between the pre‐COVID period (2010–2019) and the COVID period (2020–2023) (Figure 4). For IHD, the global ASDR declined during 2010–2019 (AAPC −1.35% per year, 95% CI −1.74 to −0.96) but largely plateaued during 2020–2023 (AAPC 0.06% per year, 95% CI −1.82 to 1.99). A similar pattern was observed for ASDALY (2010–2019: −1.30%, 95% CI −1.66 to −0.95; 2020–2023: 0.37%, 95% CI −1.87 to 2.66), suggesting an attenuation of the prepandemic downward trend. In contrast, IS continued to show declining mortality, although with some slowing: global ASDR decreased at −1.96% per year (95% CI −2.39 to −1.54) in 2010–2019 and −1.48% per year (95% CI −2.83 to −0.11) in 2020–2023. For IS ASDALY, the decline also attenuated (2010–2019: −1.68%, 95% CI −2.01 to −1.34; 2020–2023: −1.12%, 95% CI −2.44 to 0.23). SDI‐stratified patterns were heterogeneous, with high‐SDI settings generally maintaining declining trends, whereas several non–high‐SDI strata showed greater attenuation or reversal, particularly for IHD (Figure 4; Supporting Table S4).

#### 3.5.2. Observed vs. Expected (Counterfactual Deviation)

**FIGURE 5:**
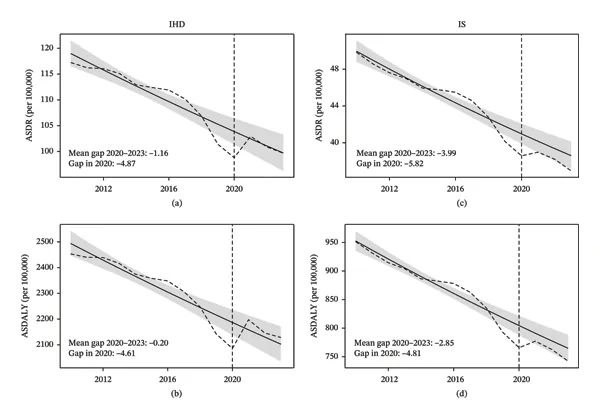
Counterfactual deviations in global age‐standardized death and DALY rates during the COVID‐19 period. Observed (dashed line) and counterfactual expected (solid line) trends are shown for ischemic heart disease (IHD) and ischemic stroke (IS) at the global level. Panels show: (a) age‐standardized death rate (ASDR) for IHD; (b) age‐standardized DALY rate (ASDALY) for IHD; (c) ASDR for IS; and (d) ASDALY for IS. Expected values were extrapolated from the pre‐COVID trend estimated over 2010–2019, with the shaded band indicating the 95% confidence interval for the expected series. The vertical dashed line marks 2020. Panel annotations report the mean relative gap over 2020–2023 and the relative gap in 2020, defined as (observed − expected)/expected × 100%; negative values indicate observed rates lower than expected. Abbreviations: ASDR, age‐standardized death rate; ASDALY, age‐standardized DALY rate; DALY, disability‐adjusted life year.

Counterfactual analyses extrapolating pre‐COVID trends (2010–2019) suggested that global observed rates during 2020–2023 were modestly lower than expected for both outcomes, with larger deviations for IS than for IHD (Figure 5). The mean relative gaps (observed−expected)/expected over 2020–2023 were −1.16% for IHD ASDR and −0.20% for IHD ASDALY, compared with −3.99% for IS ASDR and −2.85% for IS ASDALY (Supporting Table S5). Deviations were most pronounced in 2020 (IHD: ASDR −4.87%, ASDALY −4.61%; IS: ASDR −5.82%, ASDALY −4.81%), after which IHD largely converged back toward the counterfactual trajectory (2021–2023 gaps close to zero). In contrast, IS generally remained below the expected trajectory across 2020–2023, as reflected in the year‐specific deviations provided in Supporting Table S6 (Figure 5).

### 3.6. Risk Factors: Regional/Sex Heterogeneity and Profile Shifts

#### 3.6.1. Regional and Sex Heterogeneity in 2023

**FIGURE 6:**
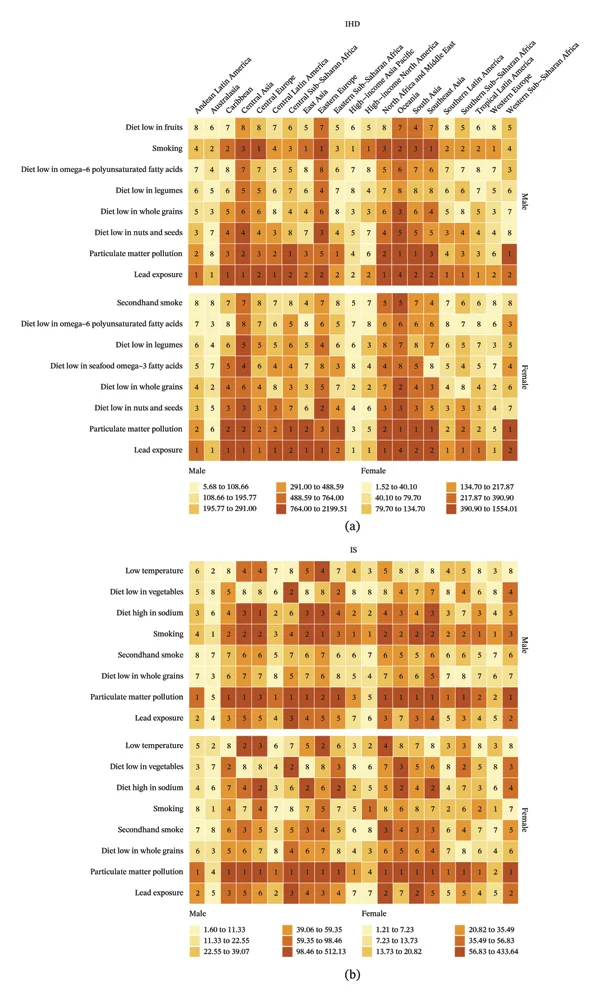
Regional‐ and sex‐specific patterns of leading attributable risk factors for ischemic heart disease and ischemic stroke in 2023. Heatmaps display the top eight attributable risk factors across GBD regions in 2023, stratified by sex. Panels show: (a) leading attributable risk factors for ischemic heart disease (IHD) and (b) leading attributable risk factors for ischemic stroke (IS). Cell colors represent the attributable DALY rate per 100,000 population, and the overlaid numbers indicate the within‐region rank [1–8] of each risk factor for the corresponding disease and sex. Risk factors shown are those with the highest attributable DALY rates within each disease–sex stratum. Abbreviations: DALY, disability‐adjusted life year; GBD, Global Burden of Disease.

In 2023, the leading attributable risk factors for both IHD and IS showed marked heterogeneity across GBD regions and by sex (Figure 6). For IHD, dietary risks consistently featured among the dominant contributors across regions—particularly diets low in fruits, whole grains, nuts and seeds, legumes, and omega‐6 polyunsaturated fatty acids—alongside tobacco‐related risks. Sex‐specific patterns were evident, with smoking prominently ranked among leading risks in males, whereas secondhand smoke was more prominent among females. Environmental exposures, notably particulate matter pollution and lead exposure, also ranked among the top contributors in many regions, frequently appearing within the highest‐ranked positions. For IS, the risk‐factor pattern differed, with particulate matter pollution and tobacco‐related risks commonly appearing among the leading contributors across regions, together with dietary/metabolic risks such as diet high in sodium, diet low in vegetables, and diet low in whole grains; low temperature emerged as an additional leading risk in several regions. Complete regional‐ and sex‐specific rankings and attributable DALY‐rate estimates are provided in Supporting Table S7.

#### 3.6.2. Global Risk‐Profile Shifts Before vs. During COVID‐19

**FIGURE 7:**
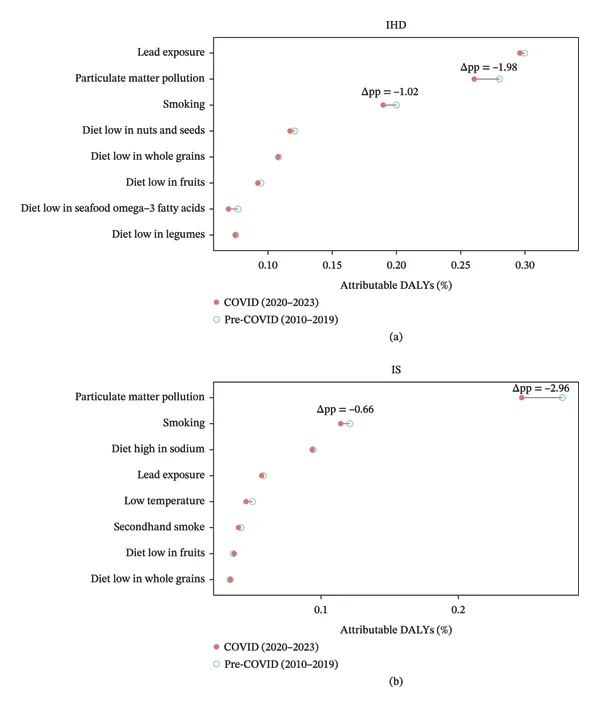
Global shifts in attributable risk‐factor profiles for ischemic heart disease and ischemic stroke before and during the COVID‐19 period. Dumbbell plots compare the mean attributable DALY percentage for leading risk factors in the pre‐COVID period (2010–2019) and the COVID period (2020–2023) at the global level (both sexes) for (a) ischemic heart disease (IHD) and (b) ischemic stroke (IS). Open circles represent pre‐COVID estimates, and filled circles represent COVID‐period estimates; horizontal connectors indicate the direction and magnitude of change between periods. Annotated values (Δpp) denote percentage‐point changes in attributable DALY percentage (COVID minus pre‐COVID) for selected risk factors. Abbreviations: DALY, disability‐adjusted life year; pp, percentage points.

Δpp denotes percentage‐point changes in attributable DALY percentage (COVID minus pre‐COVID). At the global level, the overall attributable risk profiles remained broadly stable between the pre‐COVID period (2010–2019) and the COVID period (2020–2023), with only modest shifts in the relative contributions of individual risks (Figure 7; Supporting Table S8). For IHD, the largest decreases in attributable DALY percentage were observed for particulate matter pollution (Δpp = −1.98) and smoking (Δpp = −1.02), followed by diet low in seafood omega‐3 fatty acids (Δpp = −0.75); the rank ordering was largely preserved, with only minor rank swapping among lower‐ranked dietary risks. For IS, the most pronounced reduction was again observed for particulate matter pollution (Δpp = −2.96), with smaller decreases for smoking (Δpp = −0.66) and low temperature (Δpp = −0.47). Other risk factors exhibited minimal changes in attributable share across periods, indicating limited reordering of the leading global risk profile during the COVID period.

### 3.7. Forecasting Scenarios

**FIGURE 8:**
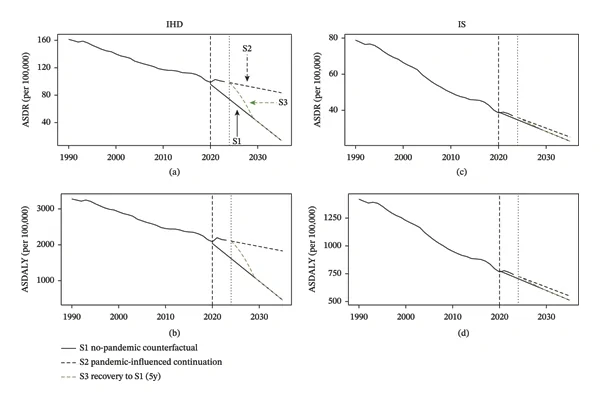
ARIMA‐based scenario forecasts of global age‐standardized death and DALY rates for ischemic heart disease and ischemic stroke. Observed trajectories from 1990 to 2023 are shown as solid black lines for (a) IHD ASDR; (b) IHD ASDALY; (c) IS ASDR; and (d) IS ASDALY (rates per 100,000 population). Three forward‐looking scenarios are overlaid: S1 (no‐pandemic counterfactual) extrapolates the pre‐COVID trend estimated using 2010–2019 data; S2 (pandemic‐influenced continuation) extrapolates the trajectory estimated using the full series 2010–2023 (including the pandemic period); and S3 (recovery to S1, 5 years) assumes a gradual transition from S2 back toward S1 over 2024–2028, converging with S1 thereafter. The dashed vertical line indicates 2020 (COVID‐19 onset), and the dotted vertical line indicates 2024 (start of the recovery scenario). Abbreviations: ARIMA, autoregressive integrated moving average; IHD, ischemic heart disease; IS, ischemic stroke; ASDR, age‐standardized death rate; ASDALY, age‐standardized DALY rate.

ARIMA‐based scenario forecasts were used to project global ASDR and ASDALY for IHD and IS through 2035 (Figure 8). The main analysis adopted a pre‐COVID training window aligned with the segmented‐trend and counterfactual evaluations, with S1 fitted to 2010–2019 and S2 fitted to 2010–2023. Across scenarios, both outcomes were projected to continue declining, with clearer scenario separation after 2024. Under the no‐pandemic counterfactual scenario (S1), global IHD ASDR was projected to decrease to 41.3 (95% prediction interval 0.0–100.0) in 2030 and 13.9 (0.0–114.9) in 2035, while IS ASDR was projected to decrease to 28.3 (23.3–33.2) in 2030 and 22.9 (16.9–28.9) in 2035 (Table 2). For overall burden, IHD ASDALY was projected to decline to 985.4 (0.0–2100.1) in 2030 and 459.5 (0.0–2376.2) in 2035, and IS ASDALY to 598.9 (521.1–676.6) in 2030 and 510.7 (417.0–604.5) in 2035 (Table 2).

**TABLE 2:** ARIMA‐based projections of global age‐standardized death and DALY rates for ischemic heart disease and ischemic stroke in 2030 and 2035 under two scenarios.

Outcome (rate per 100,000)	Disease	2030 S1	2030 S2	2035 S1	2035 S2
ASDR	IHD	41.3 (0.0–100.0)	90.2 (79.0–101.5)	13.9 (0.0–114.9)	83.5 (68.7–98.2)
ASDR	IS	28.3 (23.3–33.2)	30.1 (24.6–35.7)	22.9 (16.9–28.9)	25.2 (17.8–32.6)
ASDALY	IHD	985.4 (0.0–2100.1)	1953.7 (1696.4–2211.0)	459.5 (0.0–2376.2)	1828.8 (1491.9–2165.7)
ASDALY	IS	598.9 (521.1–676.6)	629.7 (561.6–697.7)	510.7 (417.0–604.5)	549.2 (460.1–638.3)

*Note:* Values are presented as point forecasts (95% prediction intervals) of age‐standardized rates per 100,000 population. S1 (no‐pandemic counterfactual) extrapolates the pre‐COVID trajectory estimated using 2010–2019 data, whereas S2 (pandemic‐influenced continuation) extrapolates the trajectory estimated using the full series 2010–2023 (including the pandemic period). Lower bounds of prediction intervals were constrained to be non‐negative (truncated at 0).

Relative to the counterfactual, the pandemic‐influenced continuation scenario (S2) yielded consistently higher projected levels for both diseases and both metrics at the same time points. For example, in 2030, IHD ASDR under S2 was 90.2 (79.0–101.5) compared with 41.3 (0.0–100.0) under S1, and IS ASDR under S2 was 30.1 (24.6–35.7) compared with 28.3 (23.3–33.2) under S1 (Table 2). By 2035, projections remained higher under S2 (IHD ASDR 83.5 (68.7–98.2) vs. 13.9 (0.0–114.9) under S1; IS ASDR 25.2 (17.8–32.6) vs. 22.9 (16.9–28.9) under S1), with similar patterns for ASDALY (Table 2). The recovery scenario (S3) in Figure 8 illustrates an intermediate trajectory that gradually converges toward the counterfactual path over a 5‐year recovery window starting in 2024. Sensitivity analyses using a longer training window starting in 1990 are reported in Supporting Figure S1 and Supporting Table S9. Prediction intervals were particularly wide for some long‐horizon IHD projections under the 2010‐start S1 scenario, while the 1990‐start sensitivity analysis yielded different projected trajectories, indicating substantial uncertainty at longer horizons and sensitivity to training‐window specification.

## 4. Discussion

### 4.1. Summary of Key Findings

In this comprehensive GBD 2023–based assessment of IHD and IS from 1990 to 2023, several overarching patterns emerged. First, substantial country‐level heterogeneity persisted in 2023, with a higher upper‐end mortality burden for IHD than for IS (maximum ASDR 441.0 vs. 182.5 per 100,000). Second, age‐standardized incidence, mortality, and DALY rates generally declined over the long term, but the magnitude of improvement differed by metric and by SDI strata; notably, IHD mortality and DALY rates increased in low‐SDI settings despite pronounced declines in high‐SDI settings. Third, mortality burden remained highly concentrated in older adults, with those aged ≥ 75 years accounting for the greatest age‐specific death rates in both diseases. Fourth, the COVID‐19 period functioned as a measurable “stress test” of prior progress: IHD declines attenuated to near‐plateau during 2020–2023, while IS continued to decline but at a slower pace. Finally, leading attributable risks showed marked regional/sex heterogeneity yet relatively stable global composition between pre‐COVID and COVID periods, and scenario‐based forecasts suggested continued declines through 2035 with systematically higher projections under pandemic‐influenced trajectories relative to counterfactual scenarios.

### 4.2. Long‐Term Progress With Widening SDI Inequalities

Across 1990–2023, age‐standardized rates for IHD and IS declined globally on average, consistent with prior GBD syntheses reporting long‐term reductions in age‐standardized cardiovascular mortality, albeit with substantial geographic heterogeneity [3, 18]. Notably, the pattern of improvement differed between diseases: IHD showed broadly concordant declines across age‐standardized incidence, mortality, and DALY rates, whereas for IS the relative reductions in mortality and DALYs exceeded the decline in incidence. This divergence may reflect that advances in acute stroke care and secondary prevention have reduced case fatality and disability burden more rapidly than primary prevention has reduced incident events [4, 19, 20]. Importantly, in our analysis, changes after 2019 were smaller in magnitude than long‐term declines, indicating a more modest recent pace of improvement in age‐standardized mortality.

These rate‐based gains coexisted with rising absolute burden, with deaths and DALYs increasing for both IHD and IS, particularly IHD (Supporting Table S12). This “progress paradox” may reflect the influence of population growth and aging, although we did not formally decompose demographic and epidemiological contributions.

However, these aggregate gains concealed widening development‐related inequities, most prominently for IHD. A striking reversal was observed across SDI strata: High‐SDI settings experienced substantial long‐term reductions in IHD mortality, whereas low‐SDI settings showed increases in IHD mortality and DALY burden over the same period. Similar SDI‐graded disparities in IHD burden have also been highlighted in prior GBD‐based syntheses, which emphasize that cross‐location differences in cardiovascular burden reflect both heterogeneity in risk‐factor exposure and uneven access to effective prevention and care [3, 21, 22]. In contrast, IS mortality declined across all SDI strata, but improvements were markedly larger in high‐SDI settings than in low‐SDI settings, consistent with persistent gradients in potentially preventable mortality documented in global stroke burden assessments [4]. Taken together, these findings support an “implementation gap” interpretation: Durable declines are most achievable where primary prevention, timely acute care, and long‐term secondary prevention can be delivered consistently, whereas settings facing rising cardiometabolic risks and constrained health‐system capacity may experience stagnation or reversal—particularly for IHD [3, 23].

### 4.3. COVID‐19 Period as a Stress Test of Trends

Using complementary segmented‐trend and counterfactual approaches, we found that the COVID‐19 period was associated with a measurable attenuation of recent burden trajectories, more pronounced for IHD than for IS. In segmented analyses, the prepandemic decline in IHD mortality shifted to an overall plateau during 2020–2023, whereas IS mortality continued to decline but at a slower pace; analogous patterns were observed for DALY rates. Counterfactual extrapolation of pre‐COVID trends suggested that observed rates in 2020–2023 were modestly below expected overall, with the largest deviations occurring in 2020 and a more persistent below‐expected trajectory for IS than for IHD.

Interpretation should remain cautious given the descriptive nature of these analyses; nevertheless, the observed inflection is concordant with a growing body of evidence that the pandemic perturbed cardiovascular risk exposure and the delivery of time‐sensitive care. Studies have documented reductions in emergency presentations and hospitalizations for acute myocardial infarction consistent with care avoidance and broader disruptions, alongside measurable changes in treatment patterns and outcomes when hospital COVID‐19 burden was high [7, 8, 24, 25]. For stroke, multinational data indicate substantial declines in stroke admissions and reperfusion‐related activity (e.g., mechanical thrombectomy volume) during the pandemic, consistent with system‐level constraints and delayed or foregone care‐seeking in parts of the population [9, 26, 27]. Importantly, both acute coronary syndromes and IS are highly time‐sensitive conditions, although pandemic‐related disruptions may have affected their care pathways differently. In parallel, direct effects of SARS‐CoV‐2 infection may have contributed by increasing short‐ and medium‐term cardiovascular risk among survivors, potentially affecting background event rates and post‐acute burden [10, 28]. These mechanisms—operating concurrently and heterogeneously across settings—provide plausible explanations for why IHD trajectories exhibited greater attenuation and why IS remained below its counterfactual trajectory beyond 2020. Evidence of excess cardiovascular mortality peaks during the pandemic further supports the notion that indirect and direct pathways jointly influenced cardiovascular outcomes over this period [11].

Several factors may help explain the contrasting trajectories of IHD and IS. IS entered the pandemic with a steeper prepandemic decline in mortality, which may have contributed to its continued downward trend. The greater proportional mortality reductions observed in older adults also suggest comparatively smaller gains at younger ages, consistent with recent evidence highlighting the burden of IHD in younger and middle‐aged adults [29]. In the counterfactual analysis, IHD deviations were concentrated in 2020 and subsequently converged toward expected levels, whereas IS remained below its extrapolated trajectory through 2023. This should not be interpreted as a protective pandemic effect but may reflect differences in underlying secular trends, recovery of disease‐specific care pathways, competing mortality, cause‐of‐death attribution, and uncertainty in counterfactual extrapolation. These mechanisms cannot be disentangled using aggregate GBD data.

### 4.4. Risk‐Factor Structure and Policy Implications, With Scenario‐Based Forecasts

Our risk attribution results reinforce that the dominant, modifiable drivers of IHD and IS burden remain largely structural and policy‐sensitive—most notably tobacco exposure, dietary risk patterns, and environmental hazards—despite substantial regional and sex heterogeneity in ranking [30]. This is consistent with recent GBD risk‐factor syntheses showing that a relatively stable set of behavioral, metabolic, and environmental risks continues to account for a large share of global health loss, even as exposure distributions shift across settings [6]. The modest compositional changes observed between the pre‐COVID and COVID periods therefore likely reflect incremental shifts in exposure intensity rather than a fundamental reordering of underlying determinants. In practice, this argues for sustained, population‐level prevention strategies: dietary pattern improvement (rather than single‐nutrient approaches), comprehensive tobacco control, and accelerated reduction of pollution‐related exposures [12, 31–33]. Lead exposure deserves explicit emphasis as an inequity‐amplifying environmental risk, with recent Lancet Planetary Health analyses highlighting its substantial health and economic costs and the feasibility of prevention through upstream regulation and remediation [34, 35]. Complementing these structural interventions, scenario modeling work indicates that achievable reductions in smoking prevalence can translate into large downstream mortality gains, reinforcing the value of prevention‐focused policy packages [36].

The country‐ and territory‐specific estimates in Supporting Tables S10 and S11 provide a practical framework for national benchmarking. The greater frequency of adverse trajectories for IHD than for IS, particularly over 2019–2023, suggests that countries may face different combinations of persistently high burden, inadequate long‐term improvement, and recent loss of progress. These profiles can help inform priorities for risk‐factor control, continuity of preventive care, timely acute cardiovascular services, and health‐system resilience, while requiring interpretation in the context of local risk profiles, health‐system capacity, and resource constraints. In low‐SDI settings, priorities include improving access to essential cardiovascular care, integrating risk‐factor detection and management into primary care, and strengthening community‐based prevention, with age‐ and sex‐responsive approaches across the life course [37].

Our scenario‐based projections extend these implications to 2035. Although continued declines in ASDR and ASDALY remain plausible, divergence across scenarios and wide prediction intervals indicate substantial uncertainty in future trajectories. The 2010‐start analysis is retained as the primary framework because it reflects the more recent prepandemic trajectory and aligns with our pandemic‐period analyses, whereas the 1990‐start analysis serves as a sensitivity analysis of longer‐term trends and training‐window dependence. Accordingly, the S1–S2 divergence should be interpreted as conditional scenario uncertainty and potential “headroom” for improvement, rather than as deterministic predictions or formal best‐ and worst‐case bounds. A pragmatic response—particularly in settings where IHD burden has risen—requires a dual strategy: (1) population‐wide risk reduction (tobacco control, healthier food environments, and pollution mitigation) and (2) equitable scale‐up of evidence‐based cardiovascular prevention and long‐term management in primary care and secondary prevention pathways [13, 22, 38, 39]. The latter includes systematic detection and control of hypertension and dyslipidemia and sustained delivery of proven therapies for chronic coronary disease and atherosclerotic cardiovascular disease, alongside service models that protect medication continuity during system‐wide shocks [13, 40].

### 4.5. Strengths and Limitations

This study has several strengths. It provides a unified, multimetric comparison of IHD and IS burden over more than 3 decades, integrating spatial mapping, SDI‐stratified inequality assessment, age‐ and sex‐specific characterization, and comparative risk attribution. The COVID‐19 assessment leveraged two complementary analytical lenses—segmented trend estimation and counterfactual deviation—offering convergent evidence on pandemic‐era inflections in burden trajectories. Finally, scenario‐based forecasting through 2035 provides a forward‐looking extension that is directly relevant for public health planning and resource allocation.

Several limitations should be considered when interpreting the findings. First, the analysis is ecological and descriptive; observed associations across SDI strata or regions cannot establish causal relationships at the individual level, and within‐country heterogeneity is not captured by national averages. Second, GBD estimates are model‐based syntheses that depend on the availability and quality of underlying data (e.g., cause‐of‐death certification, surveillance, and covariate inputs), which vary across settings and may influence cross‐country comparability despite standardized methods and UIs. Third, comparative risk assessment estimates rely on assumptions regarding exposure distributions, relative risks, mediation structures, and independence among risks; overlapping pathways can complicate attribution and interpretation of small compositional changes. Age‐period‐cohort analyses were not performed; future studies could use this framework to further disentangle age, period, and cohort‐specific contributions to the observed divergence in IHD trends across SDI strata. Fourth, counterfactual analyses assume that pre‐COVID trends (2010–2019) would have continued in the absence of the pandemic, an assumption that may not fully account for concurrent secular changes or policy shifts. In addition, although SARS‐CoV‐2 infection may directly increase cardiovascular risk, the present ecological analysis did not formally quantify the association between infection‐related mortality and IHD or IS outcomes. Disentangling direct infection‐mediated effects from health‐system disruption, competing mortality, and changes in cause‐of‐death attribution will require dedicated analyses using harmonized infection‐burden data and more granular longitudinal data. Finally, ARIMA‐based projections are sensitive to training‐window choice and future structural breaks; differences between the 2010‐ and 1990‐start analyses and wide long‐horizon prediction intervals therefore support interpreting them as conditional scenarios rather than precise forecasts.

### 4.6. Conclusions

From 1990 to 2023, the global age‐standardized burden of IHD and IS generally declined, yet progress has been uneven and accompanied by pronounced and, for IHD, reversed SDI gradients. Mortality remains highly concentrated in older adults, underscoring the growing importance of healthy aging and secondary prevention. The COVID‐19 period was associated with a marked attenuation of prepandemic declines—most evident for IHD—highlighting the vulnerability of long‐term cardiovascular gains to system‐wide disruptions. Leading attributable risk structures remained broadly stable, emphasizing that sustained control of tobacco exposure, dietary risks, and environmental pollutants remains central to future reductions. Forecast scenarios suggest continued improvement through 2035 but also indicate that pandemic‐influenced trajectories could translate into a persistently higher burden, particularly if inequities in prevention and care are not addressed.

## Author Contributions

Haohao Chen: conceptualization; methodology; data curation; formal analysis; software; visualization; writing–original draft.

Boya Hu: data curation; formal analysis; writing–review and editing.

Ziwen Xiong: data curation; visualization; writing–review and editing.

Mengting Zhao: data curation; visualization; writing–review and editing.

Bingxuan Chen: validation; visualization; writing–review and editing.

Jinhua Yang: validation; writing–review and editing.

Yali Wang: data curation; writing–review and editing.

Ying Zeng: conceptualization; supervision; writing–review and editing.

Fenfei Gao: methodology; supervision; project administration; writing–review and editing.

De Cai: conceptualization; supervision; project administration; writing–review and editing.

## Funding

This study was funded by the Chronic Disease Management Research Project of the National Health Commission Capacity Building and Continuing Education Center (GWJJMB202510021013), the Special Research Project of the National Health Commission Capacity Building and Continuing Education Center (GWJJZX20251008008), and the Guangdong Province Science and Technology Special Fund Project (210716096900467).

## Ethics Statement

This study used publicly available, de‐identified, aggregated estimates from the Global Burden of Disease (GBD) 2023 study. As no individual‐level identifiable data were accessed, ethical approval and informed consent were not required.

## Conflicts of Interest

The authors declare no conflicts of interest.

## Supporting Information

Additional supporting information can be found online in the Supporting Information section.

## Supporting information


**Supporting Information** Supporting Figure 1. ARIMA‐based scenario forecasts of global age‐standardized death and DALY rates for ischemic heart disease and ischemic stroke (1990‐start training). Observed trajectories from 1990 to 2023 are shown as solid black lines for (a) ischemic heart disease (IHD) ASDR; (b) IHD ASDALY; (c) ischemic stroke (IS) ASDR; and (d) IS ASDALY (rates per 100,000 population). Three forward‐looking scenarios are overlaid: S1 (no‐pandemic counterfactual) extrapolates the pre‐COVID trend estimated using 1990–2019 data; S2 (pandemic‐influenced continuation) extrapolates the trajectory estimated using the full series 1990–2023 (including the pandemic period); and S3 (recovery to S1, 5 years) assumes a gradual transition from S2 back toward S1 over 2024–2028, converging with S1 thereafter. The dashed vertical line indicates 2020 (COVID‐19 onset), and the dotted vertical line indicates 2024 (start of the recovery scenario). Supporting Table S1. Annual age‐standardized incidence, death, and DALY rates for ischemic heart disease and ischemic stroke at the global level and across SDI quintiles, 1990–2023. Supporting Table S2. Age‐standardized DALY rates (ASDALY) for ischemic heart disease and ischemic stroke by SDI quintile in 1990, 2019, 2020, and 2023, and percentage changes over 1990–2023 and 2019–2023. Supporting Table S3. Global age‐ and sex‐specific death and DALY rates for ischemic heart disease and ischemic stroke across adult age groups in 1990, 2019, 2020, and 2023. Supporting Table S4. Segmented‐trend estimates (AAPC) for age‐standardized incidence, death, and DALY rates of ischemic heart disease and ischemic stroke at the global level and across SDI quintiles, pre‐COVID (2010–2019) versus COVID period (2020–2023). Supporting Table S5. Counterfactual deviations between observed and expected age‐standardized rates during 2020–2023 (mean gaps and 2020 gaps) for ischemic heart disease and ischemic stroke at the global level and across SDI quintiles. Supporting Table S6. Year‐specific counterfactual deviations between observed and expected age‐standardized rates for ischemic heart disease and ischemic stroke, 2020–2023, at the global level and across SDI quintiles. Supporting Table S7. Regional‐ and sex‐specific rankings and attributable DALY rates of leading risk factors for ischemic heart disease and ischemic stroke in 2023. Supporting Table S8. Attributable DALY percentages for leading risk factors in 2010–2019 and 2020–2023 and percentage‐point changes (Δpp) for ischemic heart disease and ischemic stroke by SDI quintile. Supporting Table S9. ARIMA‐based projections of global age‐standardized death and DALY rates for ischemic heart disease and ischemic stroke in 2030 and 2035 under two scenarios (1990‐start sensitivity analysis). Supporting Table S10. Country‐specific age‐standardized death rates for ischemic heart disease and ischemic stroke in selected reference years and temporal changes, 1990–2023. Supporting Table S11. Country‐specific age‐standardized DALY rates for ischemic heart disease and ischemic stroke in selected reference years and temporal changes, 1990–2023. Supporting Table S12. Global absolute deaths and DALYs due to ischemic heart disease and ischemic stroke in selected reference years, 1990–2023.

## Data Availability

All data used in this study are publicly available from the Institute for Health Metrics and Evaluation (IHME) Global Burden of Disease (GBD) Results Tool (GBD 2023). The data can be accessed and downloaded via the GBD Results Tool. The analytical code used to generate the results and figures is available from the corresponding author upon reasonable request.
