# Supplementary material for: Global Burden of Ischemic Heart Disease and Ischemic Stroke, 1990–2023: Spatial Heterogeneity, SDI Inequalities, Pandemic‐Era Inflections, and Scenario Forecasts to 2035 Using GBD 2023
Source: Cardiol Res Pract. 2026 Sep 15;2026:6248551. doi: 10.1155/crp/6248551 (PMC13579108; doi:10.1155/crp/6248551)
Supplement: Supplementary file 1 [file CRP-2026-6248551-s001.zip › Supplementary Table S2.docx]

Supplementary Table S2. Age-standardised DALY rates (ASDALY) for ischemic heart disease and ischemic stroke by SDI quintile in 1990, 2019, 2020, and 2023, and percentage changes over 1990–2023 and 2019–2023.

| Disease |  | SDI group | 1990 | 2019 | 2020 | 2023 | % change (1990–2023) | % change (2019–2023) |
| --- | --- | --- | --- | --- | --- | --- | --- | --- |
| IHD |  | Global | 3277.16 (3037.13–3529.14) | 2142.37 (1972.43–2292.13) | 2086.14 (1926.46–2225.77) | 2128.58 (1939.96–2314.66) | -35.05  (-45.03– -23.79) | -0.64  (-15.36–17.35) |
|  |  | Low | 2447.21 (1910.38–3007.17) | 2557.52 (2193.42–2961.61) | 2449.94 (2076.19–2835.82) | 2626.62 (2168.82–3105.17) | 7.33  (-27.88–62.54) | 2.70  (-26.77–41.57) |
|  |  | Low-middle | 3337.74 (2693.48–3957.64) | 2852.08 (2553.56–3206.22) | 2772.97 (2427.66–3091.18) | 2851.38 (2523.62–3273.59) | -14.57  (-36.23–21.54) | -0.02  (-21.29–28.20) |
|  |  | Middle | 3130.57 (2659.88–3614.88) | 2948.94 (2650.18–3212.68) | 2859.32 (2550.54–3140.74) | 2996.44 (2654.10–3349.23) | -4.28  (-26.58–25.92) | 1.61  (-17.39–26.38) |
|  |  | High-middle | 2878.26 (2523.03–3212.34) | 2036.27 (1864.18–2180.80) | 1952.65 (1779.10–2087.29) | 2008.32 (1815.36–2180.36) | -30.22  (-43.49– -13.58) | -1.37  (-16.76–16.96) |
|  |  | High | 3423.04 (3237.95–3576.52) | 1722.28 (1613.91–1801.69) | 1699.45 (1596.34–1777.63) | 1662.85 (1550.40–1751.11) | -51.42  (-56.65– -45.92) | -3.45  (-13.95–8.50) |
| IS |  | Global | 1419.46 (1288.04–1580.63) | 792.82 (698.76–884.94) | 765.78 (678.16–849.54) | 742.32 (649.17–832.71) | -47.70  (-58.93– -35.35) | -6.37  (-26.64–19.17) |
|  |  | Low | 1084.53 (780.97–1519.61) | 987.76 (740.36–1259.19) | 950.53 (708.68–1216.25) | 936.10 (687.22–1212.86) | -13.69  (-54.78–55.30) | -5.23  (-45.42–63.82) |
|  |  | Low-middle | 1223.29 (943.22–1604.69) | 1097.84 (854.58–1367.73) | 1062.81 (839.44–1289.12) | 1034.83 (799.76–1272.80) | -15.41  (-50.16–34.94) | -5.74  (-41.53–48.94) |
|  |  | Middle | 1483.08 (1221.12–1748.93) | 1085.55 (885.89–1289.76) | 1044.51 (849.86–1246.63) | 1018.57 (832.93–1193.62) | -31.32  (-52.38– -2.25) | -6.17  (-35.42–34.74) |
|  |  | High-middle | 1429.08 (1222.97–1695.83) | 759.72 (681.51–839.32) | 716.28 (640.95–791.67) | 699.33 (610.66–791.44) | -51.06  (-63.99– -35.28) | -7.95  (-27.24–16.13) |
|  |  | High | 1449.82 (1341.32–1556.33) | 651.78 (588.21–702.60) | 637.30 (577.48–687.81) | 607.85 (543.17–662.58) | -58.07  (-65.10– -50.60) | -6.74  (-22.69–12.64) |

Note: Values are ASDALY rates per 100,000 population, with 95% uncertainty intervals (UIs) in parentheses. Percentage changes are reported as % (95% UI). Estimates are for both sexes. SDI quintiles (low, low-middle, middle, high-middle, high) were defined using the GBD SDI framework.

Abbreviations: SDI, Socio-demographic Index.
