# Supplementary material for: Global Burden of Ischemic Heart Disease and Ischemic Stroke, 1990–2023: Spatial Heterogeneity, SDI Inequalities, Pandemic‐Era Inflections, and Scenario Forecasts to 2035 Using GBD 2023
Source: Cardiol Res Pract. 2026 Sep 15;2026:6248551. doi: 10.1155/crp/6248551 (PMC13579108; doi:10.1155/crp/6248551)
Supplement: Supplementary file 1 [file CRP-2026-6248551-s001.zip › Supplementary Table S9.docx]

Supplementary Table S9. ARIMA-based projections of global age-standardised death and DALY rates for ischemic heart disease and ischemic stroke in 2030 and 2035 under two scenarios (1990-start sensitivity analysis).

| Ootcome  (rate per 100,000) | Disease | 2030 S1 | 2030 S2 | 2035 S1 | 2035 S2 |
| --- | --- | --- | --- | --- | --- |
| ASDR | IHD | 77.2  (66.1–88.4) | 86.6  (78.0–95.1) | 66.8  (53.2–80.3) | 77.2  (66.1–88.4) |
| ASDR | IS | 23.6  (16.3–30.8) | 28.2  23.6–32.8) | 16.7  (7.7–25.7) | 21.9  (15.7–28.1) |
| ASDALY | IHD | 1676.8  (1450.6–1903.1) | 1884.9  (1698.3–2071.6) | 1478.1  (1203.0–1753.2) | 1710.9  (1466.5–1955.4) |
| ASDALY | IS | 519.0  (390.1–647.8) | 602.9  (522.6–683.2) | 407.2  (245.6–568.8) | 501.3  (394.2–608.4) |

Note: Values are presented as point forecasts (95% prediction intervals) of age-standardised rates per 100,000 population. S1 (no-pandemic counterfactual) extrapolates the pre-COVID trajectory estimated using 1990–2019 data, whereas S2 (pandemic-influenced continuation) extrapolates the trajectory estimated using the full series 1990–2023 (including the pandemic period). Projections are reported for ischemic heart disease (IHD) and ischemic stroke (IS) for ASDR (age-standardised death rate) and ASDALY (age-standardised DALY rate).
