# Supplementary material for: Global Burden of Ischemic Heart Disease and Ischemic Stroke, 1990–2023: Spatial Heterogeneity, SDI Inequalities, Pandemic‐Era Inflections, and Scenario Forecasts to 2035 Using GBD 2023
Source: Cardiol Res Pract. 2026 Sep 15;2026:6248551. doi: 10.1155/crp/6248551 (PMC13579108; doi:10.1155/crp/6248551)
Supplement: Supplementary file 1 [file CRP-2026-6248551-s001.zip › Supplementary figure.pdf]

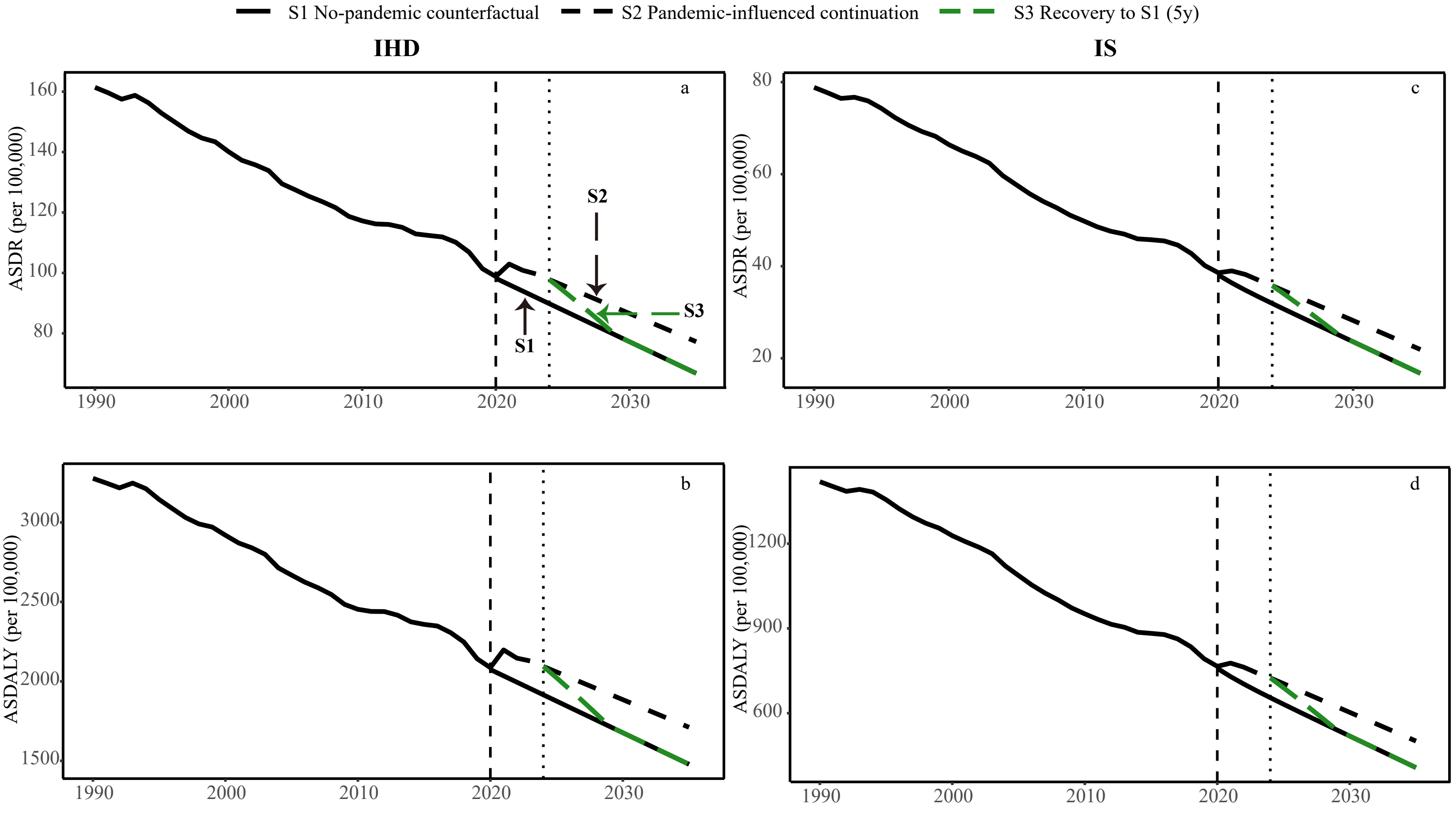

S1 No-pandemic counterfactual
S2 Pandemic-influenced continuation
S3 Recovery to S1 (5y)
IHD
IS
a
b
c
d
S1
S2
S3
ASDR (per 100,000)
ASDALY (per 100,000)
160
140
120
100
80
60
40
20
3000
2500
2000
1500
1200
900
600
1990
2000
2010
2020
2030
